# Identification of risk factors in pre-term infants with abnormal general movements

**DOI:** 10.3389/fneur.2022.850877

**Published:** 2022-11-14

**Authors:** Małgorzata Domagalska-Szopa, Andrzej Szopa, María Eugenia Serrano-Gómez, Magdalena Hagner-Derengowska, Jakub Behrendt

**Affiliations:** ^1^Department of Developmental Age Physiotherapy, Medical University of Silesia, Katowice, Poland; ^2^Department of Physiotherapy, Medical University of Silesia, Katowice, Poland; ^3^Rehabilitation and Medical Center Neuromed SC, Katowice, Poland; ^4^Facultad de Enfermería y Rehabilitación, Universidad de La Sabana, Chía, Colombia; ^5^Facultad de Psicología Ciencias de la Educación y del Deporte Blanquerna, Universidad Ramon Llull, Barcelona, Spain; ^6^Faculty of Earth Sciences and Spatial Management, University Nicolaus Copernicus, Toruń, Poland; ^7^Department of Neonatal Intensive Care, Faculty of Medical Sciences in Zabrze, Medical University of Silesia, Katowice, Poland

**Keywords:** pre-term infants, abnormal movements, risk factors, general movements, risk factors identification

## Abstract

**Introduction:**

This study aimed to investigate the relationship between prenatal, perinatal, and postnatal risk factors for neurodevelopmental impairment (NDI) with the outcomes of General Movement (GM) Assessment (GMA) in pre-term infants at 3–5 months of age. We sought to identify the risk factors associated with the predictors of psychomotor development in pre-term newborns, such as normal fidgety movements (FMs), absent FMs, or abnormal FMs, assessed during the fidgety period of motor development.

**Methods:**

The SYNAGIS program (prophylactic of Respiratory Syncytial Virus Infection) was used to identify risk factors for the development of neuromotor deficits in 164 pre-term infants who were at high risk of developing these deficits. Based on the GMA, all participants were divided into three groups of infants who presented: (1) normal FMs; (2) absent FMs; and (3) abnormal FMs.

**Results:**

The results of the current study suggest that abnormal GMs not only indicate commonly known factors like birth asphyxia (BA), respiratory distress syndrome (RDS), periventricular leukomalacia (PVL), intraventricular hemorrhage (IVH) grades 3–4, but also predict the development of motor impairments. In the present study, several specific risk factors including bronchopulmonary dysplasia (BPD), infertility treatments, maternal acute viral/bacterial infections during pregnancy, and elevated bilirubin levels were identified as attributes of an atypical fidgety movement pattern.

**Conclusions:**

Additional clinical data, such as risk factors for NDI associated with early predictors of psychomotor development in pre-term newborns, i.e., absent or abnormal FMs, may be helpful in predicting neurological outcomes in pre-term infants with developmental concerns in the 1st month of life.

## Introduction

Over the past 30 years, there has been a decrease in mortality among pre-term infants with low and very low birth weights, as well as a relative increase in the incidence of cerebral palsy (CP) in this population compared to term-born children (1, 2).

Advances in perinatal care allow most pre-term babies to survive; however, they are at increased risk of neurodevelopmental impairment (NDI) including the impairment of motor, sensory, and cognitive function (3–7). There is also a significantly higher percentage of perinatal damage to the central nervous system (CNS), leading to the development of CP, among others (8, 9). The incidence of CP in the premature infant population is relatively low (the mean prevalence of ~10%); however, the risk of other less severe motor difficulties, such as minor neurological dysfunction (MND), including impaired developmental coordination or sensory integration, is definitely higher (10). Pre-term infants are also often at higher risk of autism spectrum disorders and attention deficit hyperactivity disorder (ADHD) than full-term infants (11).

Most medical experts propose that NDI (including CP) is multifactorial in origin and occurs through a series of occurrences usually referred to as a risk factor. Because risks factors can occur during pregnancy and delivery, they are usually classified as (1) prenatal, (2) perinatal, and (3) postnatal risk factors (4–7). Prenatal risk factors are concerned with risks during pregnancy, i.e., before birth. Maternal pregnancy risk factors, risk of miscarriage, and predisposing intrauterine factors are the most common prenatal risk factors that can lead to NDI (6, 7). Perinatal risk factors define risk at delivery and include types of childbirth and delivery methods, labor and delivery complications, and childbirth complications. Several risk factors can increase a child's chances of developing NDI after birth (5, 6). Postnatal risk factors for NDI include birth asphyxia (BA), respiratory distress syndrome (RDS), bronchopulmonary dysplasia (BPD), periventricular leukomalacia (PVL), and intraventricular hemorrhage (IVH) grades 3–4 (6, 7).

The prognostic value of risk factors is essential for developmental outcomes; however, some important risk factors that are well-established in the literature may not always be statistically significant in a particular data set.

Recognition of early symptoms of later development of NDI in infancy (up to the 3rd month of life) using diagnostic methods is also challenging. General Movement (GM) Assessment (GMA) developed by Prechtl is currently one of the leading methods to identify neurological issues that may result in NDI (12–16). Among various GMs, fidgety movements (FMs) have the greatest prognostic value (12–14). Infants with normal FMs at 9–20 weeks of post-term age are more likely to have normal neurological development, whereas the absence of FMs at 3 months of post-term age is a strong predictor of an adverse neurological outcome (12–14). Absent FMs over 20 weeks of post-term age predict the development of CP by the age of 2 years, with a sensitivity of 90–98% and specificity of 90–94% in infants who were at high risk of motor impairment (17). Furthermore, the presence of abnormal FMs instead of normal FMs can be considered to be highly predictive of mild motor dysfunction and related to the presence of complex MND (18).

In general, GMA is used in academic settings to predict developmental outcomes in pre-term infants, while most pre-term infants are referred to non-academic outpatient centers (19). Therefore, additional clinical data, such as risk factors associated with atypical GMs, may help predict neurological outcomes in pre-term infants with developmental concerns in the 1st month of life. The relationship between the outcomes of GMA and premature birth (19, 20), perinatal brain lesions (20), and CP (21) has been analyzed in previous studies. Most of them confirmed that the presence of atypical GMs in the clinically relevant forms, such as definitely abnormal (DA) GM-complexity and/or absent FMs (according to the two variants of GMA), is associated with low gestational age at birth and thus a low birth weight (< 2,000 g) of infants (19–22).

Recently, several studies have investigated the association of the outcomes of both GMA, namely global (original Prechtl's method) and detailed GMA with several prenatal, perinatal, and socio-economic characteristics in the pre-term infant population. Current evidence suggests that absent FM patterns are primarily associated with the postnatal period of pre-term infants (23, 24), while maternal pregnancy risk factors are correlated to infants who present FMs, only in abnormal clinical forms (25, 26). Although the abovementioned studies indicated a few risk factors associated with the development of GMs, these findings were based on small samples from 20 to 80 infants; of these only 1–10% presented atypical GMs. According to the review findings mentioned above, there is insufficient information on the relationships between the presence of early identification of risk factors in infants and the outcomes of GMA, mainly absent FMs, which are sensitive predictors of the nature of NDI in premature babies.

Therefore, this study aimed to investigate the relationship between prenatal, perinatal, and postnatal risk factors for NDI with atypical FMs in a representative sample of pre-term infants at high risk of developing NDI. This study also aimed to identify specific risk factors associated with atypical FMs, such as absent FMs, and abnormal FMs, assessed during the fidgety period of motor development, i.e., at 3–5 months of age. Taking into account the results of previous studies, we hypothesized that (1) most of the significant risk factors related to absent FMs were associated with the postnatal period and (2) most of the significant risk factors related to abnormal FMs were associated with maternal pregnancy risk factors.

## Materials and methods

The study was approved by the Bioethical Committee of the Medical University of Silesia in Katowice under resolution No. KNW/0022/KB1/148/14, and informed consent was obtained from each participating family. The study is in accordance with the Helsinki Declaration. Parents of all children provided written informed consent before study enrolment and data collection.

Risk factors for the development of neuromotor deficits were analyzed in 164 pre-term infants with brain ultrasound abnormalities, who were under the care of the local Neonatal Counsel Clinics of Public Clinical Hospitals and qualified for the SYNAGIS program (prophylactic of Respiratory Syncytial Virus Infection) (Table 1). They participated in our previous research project on the characterization of a new posturometric test in terms of design, construct validity, and repeatability for the detection of postural control disturbances in pre-term children.

**Table 1:** Characteristics of children included in this study.

**Infant characteristics**	**Study population (*****n*** = **164)**		
	**Me**	**IQR**	**Q1–Q3**
Gestational age (weeks)	27	2	26–28
Birth weight (grams)	900	300	750–1,050
Apgar at 5th min (score)	6	3	4–7
Mother's age (year)	31.5	10	27–37
Neonatal medical index—NMI (score)	2	2	2–4
Length of time stay in hospital (days)	21	35	12–47
Sex—Girls *n* (%)	61 (37)		
Boys *n* (%)	103 (63)		

Me, median; IQR, interquartile range; Q1 and Q3, lower and upper quartiles.

Inclusion criteria for this study were as follows: (1) infants with a gestational age at birth between 25 and 32 completed weeks; (2) presence of brain ultrasound abnormalities; (3) clinical stability; and (4) approval of the examination by legal guardians. The most frequent ultrasonogram (USG) abnormalities included IVH grades 3–4, PVL grades 3 (extensive periventricular cystic lesions) and 4 (extensive cystic lesions in the deep white matter) as well as periventricular venous infarction and posthemorrhagic ventricular dilation.

Participation was excluded if they had an infection or inflammation during an examination or if they had major congenital anomalies and genetic syndromes.

To classify the participants into one of the three groups of infants: (1) with normal FMs; (2) with absent FMs; and (3) with abnormal FMs, Prechtl's method of qualitative assessment of GMs, which consisted of the video recording of GMs and GM assessment, was used.

A video recording of GMs involved filming the infant's spontaneous activity at 3 months of corrected age, according to the standard methodological principles of Prechtl's method (12–14). Three examples of GMs (at the corrected age of 12, 13, and 14 weeks) that lasted long enough to be observed and analyzed were copied and recorded separately. This resulted in an individual GM video lasting between 3 and 5 min for each period of assessment per participant.

Each video was assessed by three independent observers—physiotherapists who only knew the corrected age of the infant and had no knowledge of the risk factors for infants who participated in this study. Two of them were certified by Prechtl's method of GMA trust at a basic level and the third at an advanced level.

First, Gestalt perception was used to distinguish whether the movement pattern was a valid normal FM or an atypical FM pattern. When atypical FMs were recognized, their nature was determined and classified as absent or abnormal FMs. Both main observers independently assessed the videos. In case of disagreement with the assessments of main observers, the third observer assessed and classified the GMs.

An analysis of risk factors was conducted based on information obtained from hospital medical records. The authors developed a questionnaire for data collection (Supplementary Tables 1–3).

The questionnaire was divided into four sections. The first section collected birth data, such as gestational age, birth weight, Apgar score at 5 min, maternal age and the length of stay in the hospital, as well as the Neonatal Medical Index (NMI). The second section discusses prenatal risk factors, i.e., predisposing intrauterine factors, and the third section contains information on delivery and childbirth complications (perinatal risk factors). The fourth section discussed postnatal risk factors, i.e., neonatal complications (Supplementary Table 1).

### Statistical analysis

All statistical analyses were performed using the software package SPSS v 26.0 (IBM Corp., Armonk, NY, USA). The normality test using the Shapiro–Wilk's test was conducted on collected parametric data, which included gestational age, birth weight, Apgar score at 5 min, maternal age and the length of hospital stay, and NMI. Descriptive statistics were used to calculate the median, interquartile range, and lower and upper quartile values. Values of frequency of occurrence of particular risk factors were expressed as percentages in a contingency table.

Spearman's rank correlation coefficient (Spearman'sρ) was used to assess the correlation between gestational age, birth weight, Apgar score, maternal age, the number of overall risk factors, and particular prenatal, perinatal, and postnatal risk factors. A corrected age, calculated from the expected birth date and assessment date, was used for all infants and was rounded to weeks. Only the correlation rates (*p* < 0.05) were considered to be statistically significant. Correlation rates were calculated according to Altman recommendations: *r*_s_ < 0.2, poor; 0.21–0.4, low; 0.41–0.6, moderate; 0.61–0.8, high; and 0.81–1, very high.

To investigate the relationship between associated risk factors and predictors of psychomotor development such as normal FMs, absent FMs, and abnormal FMs, a series of chi-squared tests (χ^2^-tests) were performed for each risk factor. The strength of associations between risk factors and GM patterns was presented using the odds ratio with a 95% confidence interval (CI). For this purpose, the GM movement pattern was recoded as a nominal variable with two values (normal FMs/atypical FMs). The FM pattern was coded as normal, absent FMs as atypical, and abnormal FMs as atypical. First, the association between normal and absent FMs was tested followed by the association between normal and abnormal FMs.

To deepen the statistical inference, logistic regression analysis was performed. The dependent variable, movement pattern, was coded as 0—normal FMs, 1—absent FMs, and 2—abnormal FMs. Risk factor variables were introduced into the model as independent variables. In all the statistical tests used, test values and coefficients with values of *p* < 0.05 were considered significant.

## Results

Demographic characteristics of participants' gestational age, birth weight, Apgar score at 5 min, maternal age and the length of hospital stay, and NMI are presented in Table 1. Hence, demographic data for the study population were typical for the population of premature infants considered in previous studies (2, 27). Thanks to this, it is results obtained with other studies will be much easier.

Based on the GM assessment, all participants were divided into three infant groups:

(1) with normal FMs (*n* = 95; 58%),

(2) with absent FMs (*n* = 41; 25%), and

(3) who presented abnormal FMs instead of normal FMs (*n* = 28; 17%).

Table 2 illustrates the demographic data for the three groups of examined newborns.

**Table 2:** Characteristics of children included in an individual subgroup.

**Infant characteristics**	**Normal FMs**			**Absent FMs**			**Abnormal FMs**		
	**(*****n*** = **95)**			**(*****n*** = **41)**			**(*****n*** = **28)**		
	**Me**	**IQR**	**Q1–Q3**	**Me**	**IQR**	**Q1–Q3**	**Me**	**IQR**	**Q1–Q3**
Gestational age	28	2	27–29	27	3	25–28	26	3	25–28
Birth weight	900	285	765–1,050	970	330	720–1,050	900	300	750–1,050
Apgar at 5th min (score)	7	2	6–8	4	2	3–5	4	3	4–7
Mother's age (year)	32	10	27–37	31	8	27–35	33	9	28–37
Neonatal medical index—NMI (score)	2	2	2–4	3	2	2–4	2	2	1–3
Length of time stay in hospital (days)	22	36	12–48	21	33	14–47	14.5	9.5	10–19.5
Sex—Girls *n* (%)	50 (48)			18 (44)			10 (36)		
Boys *n* (%)	45 (42)			23 (56)			18 (64)		

Me, median; IQR, interquartile range; Q1 and Q3, lower and upper quartiles.

The inter-scorer agreement of stratification between normal and absent FMs was perfect and reached 100%; however, the recognition between normal and abnormal FMs did not show full agreement between observers and inter-scorer agreement ranged from 80 to 87%.

The relationship between gestational age, birth weight, Apgar score, maternal age, and the number of overall risk factors, in particular prenatal, perinatal, and postnatal risk factors is shown in Table 3.

**Table 3:** Spearman's rank correlation coefficient (Spearman's ρ).

	**Gestational age**	**Birth weight**	**Apgar**	**Mother's age**
Gestational age		0.82^*^	0.60	0.32
Birth weight 1	0.82^*^		0.64	0.21
Apgar at 5th min	0.60	0.64		0.18
Mother's age	0.32	0.21	0.18	
Number of overall risk factors	−0.59^*^	−0.63^*^	−0.52^*^	0.07
Number of prenatal risk factors	−0.13	−0.10	−0.32	0.04
Number of perinatal risk factors	0.17	0.19	−0.13	0.49^*^
Number of postnatal risk factors	−0.56^*^	−0.58^*^	−0.48^*^	0.02

* p < 0.05 was considered statistically significant.

A series of chi-squared tests were used to investigate the relationship of particular risk factors for NDI (prenatal, perinatal, and postnatal) with predictors of psychomotor development in pre-term newborn, namely, normal FMs, absent FMs, and abnormal FMs.

The results of the analysis of the dependence of perinatal risk factors on the occurrence of a particular pattern of GMs did not reveal statistically significant relationships (all *p* > 0.01; Table 4). The only exception there was the delivery method. The results indicated that more than 56% of children with an absent FM pattern had deliveries other than normal vaginal [mainly cesarean delivery (C-section)] and the management of complications during labor, while children with normal and abnormal FMs presented a significantly higher percentage of normal vaginal delivery (91.6 and 71.4%, respectively).

**Table 4:** Chi-squared distribution table for the association between postnatal risk factors and a particular general movement (GM) pattern.

**GMs**		**BA**		**PVL**		**High bilirubin levels**		**IVH grades 3–4**		**BPD**	
		**Absent**	**Present**	**Absent**	**Present**	**Absent**	**Present**	**Absent**	**Present**	**Absent**	**Present**
Normal	*N*	60	35	77	18	87	8	95	0	78	17
FMs	%	63.2	36.8	81.1	18.9	91.6	8.4	100	0.0	82.1	17.9
Absent	*N*	4	37	19	22	37	4	8	33	23	18
FMs	%	9.8	90.2	46.3	53.7	90.2	9.8	19.5	80.5	56.1	43.9
Abnormal	*N*	16	12	24	4	18	10	28	0	8	20
FMs	%	57.1	42.9	85.7	14.3	64.3	35.7	100	0.0	28.8	71.4
Total	*N*	80	84	120	44	142	22	131	33	109	55
	%	48.8	51.2	73.2	26.8	86.6	13.4	79.9	20.1	66.5	35.5
*χ^2^*; *p*		*χ^2^* = 33.64; *p* < 0.001		*χ^2^* = 13.53; *p* < 0.001		*χ^2^* = 26.51; *p* < 0.001		*χ^2^* = 36.34; *p* < 0.001		*χ^2^* = 35.68; *p* < 0.001	

BA, birth asphyxia; PVL, periventricular leukomalacia; IVH grades 3–4, intraventricular hemorrhage; BPD, bronchopulmonary dysplasia.

Analysis of the frequency of occurrence of risk factors in a subgroup of children with absent FMs reported that the majority of the significant risk factors related to a pattern of absent FMs were associated with a postnatal period (Table 4). They were BA, BPD, PVL, and IVH grades 3–4 (Tables 4, **6**). The highest percentage of children with BA had a pattern of absent FMs (90.2%), while in the remaining subgroups BA was significantly rare: 63.2% with normal FMs and 57.1% in children with abnormal FMs (Table 4). Children classified as absent FMs also experienced PVL significantly (53.7%) than the other two subgroups (18.9 and 17.9%, respectively) (Tables 4, **6**). Moreover, a significant dependency between GM patterns and IVH grades 3–4 was observed. Only children with absent FMs experienced intraventricular bleeding (Tables 4, **6**).

In this group of children, there was also a significant dependence between an absent FM pattern and prenatal risk factors, such as pre-disposing intrauterine factors (mostly fetal growth restriction) and maternal infections during pregnancy (Tables 5, 6). Children with absent FMs patterns were born by pregnancies with fetal growth restriction (53.6%) and infections such as toxoplasmosis, rubella, cytomegalovirus, and herpes (87.8%) during pregnancy, compared to children with normal and abnormal FMs (Tables 5, 6).

**Table 5:** Chi-squared distribution table for the association between prenatal risk factors and a particular GM pattern.

**GMs**		**IT**		**TORCH**		**EPH**		**Fetal growth restriction**	
		**Absent**	**Present**	**Absent**	**Present**	**Absent**	**Present**	**Absent**	**Present**
Normal	*N*	89	6	95	0	87	8	83	12
FMs	%	93.7	6.3	100	0.0	91.6	8.4	87.4	12.6
Absent	*N*	41	0	37	4	5	36	19	22
FMs	%	100.0	0.0	90.2	9.8	12.2	87.8	46.4	53.6
Abnormal	*N*	20	8	24	4	16	12	24	4
FMs	%	71.4	28.6	85.7	14.3	57.1	42.9	85.7	14.3
Total	*N*	150	14	156	8	108	56	126	38
	%	95.1	8.5	95.1	4.9	65.8	34.2	76.8	23.2
*χ^2^*; *p*		*χ^2^* = 18.82; *p* < 0.001		*χ^2^* = 12.31; *p* < 0.001		*χ^2^* = 20.24; *p* < 0.001		*χ^2^* = 11.19; *p* < 0.004	

IT, infertility treatments; EPH, gestosis; presence of TORCH (toxoplasmosis, rubella, cytomegalovirus, and herpes).

**Table 6:** Associations of risk factors with atypical GMs.

**Risk factors**	**Normal FMs vs. Absent FMs**		**Normal FMs vs. Abnormal FMs**	
	**OR**	**95% CI**	**OR**	**95% CI**
IT	1.000	0.256–3.900	1.167	0.196–6.938
EPH	1.000	0.165–6.052	2.667	0.396–17.977
TORCH	2.125	0.274–16.475	–	–
Fetal growth restriction	2.500	0.671–9.310	1.667	0.269–10.334
BA	10.00^*^	2.479–40.331	–	–
BPD	4.375^*^	1.295–14.778	10.500^*^	1.766–62.438
RDS	1.750	0.472–6.483	0.167	0.029–0.953
IVH grade 3 or 4	5.000^*^	1.355–18.450	–	–
PVL	1.000	0.256–3.900	–	–
High bilirubin level	1.000	0.165–1.822	24.000^*^	3.560–161.786

IT, infertility treatments; EPH, gestosis; presence of TORCH (toxoplasmosis, rubella, cytomegalovirus, and herpes); BA, birth asphyxia; BPD, bronchopulmonary dysplasia; RDS, respiratory distress syndrome; IVH grades 3–4, intraventricular hemorrhage; PVL, periventricular leukomalacia.

Data are presented as odds ratio (OR) and 95% confidence interval (CI) in brackets.

* Indicates a significant association (p < 0.05).

Analysis of the frequency of occurrence of risk factors in children with abnormal FMs presented that most of the significant risk factors were related to prenatal risk factors (Tables 5, 6). Chi-squared test results confirmed that maternal pregnancy risk factors such as infertility treatments and maternal acute viral/bacterial infections during pregnancy, occurred significantly more frequently in this group of children than in the other two groups (Tables 5, 6). In comparison to the other subgroups (17.9 and 48.9%, respectively), children classified as abnormal FMs had a higher prevalence of BPD (71.4%) (Tables 5, 6). In addition, more than half of infants with abnormal FMs were observed to have elevated bilirubin levels, while ~90% of infants with other GM patterns were not confirmed by this factor (Tables 4, 6).

The results of the analysis of the dependence of other prenatal, perinatal, and postnatal risk factors on the occurrence of a specific GM pattern showed no statistically significant relationships (all *p* > 0.01). A logistic regression analysis showed no significance of the regression model χ^2^(1) = 1.60; *p* = 0.206.

## Discussion

The present study sought to identify risk factors associated with atypical FMs, which have been widely recognized as reliable predictors of psychomotor developmental disorders in pre-term newborns. The obtained findings seem to confirm that risk factors suspected of influencing particular atypical FMs, such as absent FMs and abnormal FMs instead of normal FMs in pre-term infants at 3–5 months of age, can be identified (Tables 4, 6).

In our sample including infants with a pattern of absent FMs, most of the significantly related risk factors were primarily associated with the postnatal period of pre-term infants; however, they have also experienced an impact on some of the prenatal risk factors (Tables 5, 6). Infants having postnatal risk factors such as BA, PVL, and IVH grades 3–4 had a higher risk of occurrence of absent FMs than others who had normal FMs and those who had abnormal FMs instead of normal ones (Tables 5, 6). However, those exposed to absent FMs were also more frequent if they came from a pregnancy with fetal growth restriction and the presence of TORCH (toxoplasmosis, rubella, cytomegalovirus, and herpes).

Our study revealed that maternal pregnancy risk factors, such as infertility treatments and maternal acute viral/bacterial infections during pregnancy, occurred more frequently in infants who presented FMs but in abnormal clinical forms. Although postnatal risk factors were not dominant in infants classified as having abnormal FMs, several postnatal complications, such as BPD and elevated bilirubin levels, were noted.

Apart from the fact that the abovementioned findings support our hypothesis, assuming that most postnatal risk factors are associated with absent FMs, our results are partially consistent with reports from other studies pointing to several postnatal risk factors that are responsible for absent FMs. Studies by Hitzert et al. (24) and Zang et al. (23) showed that both forms of absent FMs (DA GM-complexity and absent FMs) were associated with neonatal complications such as RDS and BPD. Moreover, van Iersel et al. demonstrated a significant relationship between perinatal asphyxia and abnormal FMs (28). Interestingly, several other studies have shown that absent FMs at 3–5 months of corrected age are associated with maternal smoking during pregnancy (29–32).

Likewise, our findings on the association of risk factors such as BPD and elevated bilirubin levels with the development of abnormal FMs are consistent with those of several previous studies that found a significant relationship between mildly impaired GMs, that is, mildly abnormal (MA) GM-complexity and abnormal FMs, and perinatal risk factors. In two studies, Lunsing et al. (26) showed that both MA and abnormal FMs were specifically associated with moderate hyperbilirubinemia.

An interesting study of a Dutch population showed that mildly impaired FMs were associated with suboptimal socioeconomic background, such as advanced maternal age, low maternal education, and non-native ethnicity (32). There are limited number of reports that the association between GMA outcomes and perinatal risk factors reported in the existing literature may be inconclusive due to small sample sizes in previous studies (25, 26, 32, 33), or selective samples: (1) pre-term infants with moderately low birth weight; (2) very low birth weight (32, 33); or (3) extremely low birth weight (34–37), where the prevalence of abnormal GMs, that is, DA GM complexity and abnormal FMs, as well as MA FMs, that is, MA GM-complexity and abnormal FMs, did not exceed 3% of the target population. To our knowledge, except for the study by Zang et al. (23) of 104 pre-term infants (77% with normal FMs and 23% with absent FMs), our study was the second largest number of pre-term infants with 95 pre-term infants (58% with normal FMs, 25% with absent FMs, and 17% with abnormal FMs), and the only study to assess such a large sample size of infants with atypical FMs. This may explain why our results are comparable.

In addition, our findings supported the hypothesis about a strong relationship between gestational age, birth weight, and Apgar score with the total number of risk factors, as well as the number of particular risk factors (Table 3). These findings revealed that newborns with lower age, birth bodyweight, and Apgar score had a greater number of total risk factors, especially postnatal factors. The results of the present study on the frequency of prenatal, perinatal, and postnatal risk factors in the development of NDI in prematurely born children were also confirmed in the subject literature (35–37). Regarding risk factors, the literature indicates that the main factors related to NDI are low gestation age and low birth bodyweight. Our study confirmed a relationship between gestation age, birth weight, and Apgar score, despite the fact that it seems obvious given the shortened and impaired intrauterine growth in pre-term infants (Table 3). According to many authors, low birth weight and short gestational age are the main factors influencing neurological deficits (38–40).

The main finding of the present study was the identification of particular risk factors in pre-term infants with absent FMs and abnormal FMs. Although we only addressed the recognition of risk factors associated with atypical FMs not with the final neurological outcomes, it is important to remember that the clinical form of both atypical FMs is a strong predictor of adverse neurological outcomes.

According to Prechtl's method, the absence of FMs during the fidgety period of motor development is a strong predictor of CP. Therefore, it can be assumed that a group of risk factors associated with absent FMs, such as fetal growth restriction and the presence of TORCH (toxoplasmosis, rubella, cytomegalovirus, and herpes) during pregnancy, as well as the prevalence of BA, PVL, and IVH grades 3–4, can indicate the risk of adverse neurological outcomes in pre-term infants, including CP.

De Jesus et al. indicated that BA and factors related to the duration of mechanical ventilation used, BPD, sepsis, IVH grades 3–4, and hydrocephalus were the main factors, and statistically had the greatest impact on the appearance of CP (36). Another study concluded that CNS complications, such as the degree of intraventricular bleeding and PVL, had a significant impact on the development of neurodevelopmental disorders in the pre-term infant population (41). The results of several other studies also indicate that some prenatal factors, such as intrauterine growth retardation and the presence of TORCH infections (toxoplasmosis, rubella, cytomegalovirus, and herpes), influence the development of NDI (6, 41).

On the other hand, our findings enabled us to identify risk factors associated with abnormal GMs in pre-term infants during the fidgety period in development, i.e., at 3–5 months of corrected age. Our findings have also revealed that maternal pregnancy risk factors, such as infertility treatments and maternal acute viral/bacterial infections during pregnancy, occur more frequently in children classified as abnormal FMs. These children also more often had BPD and elevated bilirubin levels. Although abnormal FMs are less predictive of neurological outcome than absent FMs, they do predict the development of mild neurological impairments at 2–3 years of age (42). As shown in several studies, the presence of abnormal FMs instead of normal FMs is linked to MND [e.g., (4–6)] and can predict the development of eye–hand coordination, hearing and speech disturbances, and attention problems and aggressive behavior at school age (18, 43–45).

## Summary

The main findings of the current study are the assignment of the specific prenatal, perinatal, and postnatal risk factors for two types of atypical GMs in pre-term infants, namely absent FMs and abnormal FMs.

The findings of this study partially supported two initial hypotheses. Based on these obtained results, it can be summarized that absent FMs are mainly associated with risk factors related to postnatal period like BA, RDS, BPD, PVL, and IVH grades 3–4. Meanwhile, most of the significant risk factors related to abnormal FMs are associated with maternal pregnancy risk factors, such as infertility treatment and maternal acute viral/bacterial infections during pregnancy.

Findings on the predictive value of specific risk factors for NDI suggest that absent FMs are related not only to previously reported risk factors like BA, RDS, BPD, and maternal smoking during pregnancy, but also to several specific risk factors such as PVL and IVH grades 3–4, as well as fetal growth restriction and the presence of TORCH. The obtained results not only endorsed that BPD and elevated bilirubin levels were related to abnormal FMs but also indicated several other specific risk factors including infertility treatments and maternal acute viral/bacterial infections during pregnancy, which were identified as attributes of abnormal FMs.

## Strengths and limitations

We focused on two unique groups of pre-term infants: those with absent FMs and those with abnormal FMs. The major strength of this study was that the target populations were larger than those previously studied. Infants included in this study are now 1–3 years old; hence, their long-term neurodevelopmental outcomes are not yet fully known. The main limitation of this study was that risk factors were assigned to the outcome of GMA at 3 months of age but not to the final neurodevelopmental outcomes. A follow-up study of the examined population of 3–7 years of age is now planned.

## Conclusions

Knowledge of the risk factors for NDI associated with the early predictors of psychomotor development in pre-term newborns, namely normal FMs, absent FMs, or abnormal FMs assessed at 3–5 months of age may improve counseling for long-term neurodevelopmental outcomes. A follow-up examination is necessary to assess long-term motor and cognitive development outcomes in children.

## Data availability statement

The raw data supporting the conclusions of this article will be made available by the authors, without undue reservation.

## Ethics statement

The studies involving human participants were reviewed and approved by Ethical Committee of the Medical University of Silesia in Katowice. Written informed consent to participate in this study was provided by the participants' legal guardian/next of kin.

## Author contributions

MD-S: conceptualization. MD-S and AS: methodology, validation, and formal analysis. MD-S, AS, MS-G, and JB: investigation. MD-S, AS, and MH-D: data curation. MD-S, AS, and MS-G: writing—original draft preparation. MD-S, AS, MS-G, MH-D, and JB: writing—review and editing. All authors read the approved the version of the manuscript submitted.

## Conflict of interest

The authors declare that the research was conducted in the absence of any commercial or financial relationships that could be construed as a potential conflict of interest.

## Publisher's note

All claims expressed in this article are solely those of the authors and do not necessarily represent those of their affiliated organizations, or those of the publisher, the editors and the reviewers. Any product that may be evaluated in this article, or claim that may be made by its manufacturer, is not guaranteed or endorsed by the publisher.

## References

[1] Patra A Huang H Bauer JA Giannone PJ. Neurological consequences of systemic in ammation in the premature neonate. Neural Regen Res. (2017) 12:890–6. 10.4103/1673-5374.208547 28761416 PMC5514858

[2] Aylward GP. Neurodevelopmental outcomes of infants born prematurely. J Dev Behav Pediatr. (2005) 26:427–40. 10.1097/00004703-200512000-00008 16344661

[3] Spittle AJ Morgan C Olsen JE Novak I Cheong JLY. Early diagnosis and treatment of cerebral palsy in children with a history of preterm birth. Clin Perinatol. (2018) 45:409–20. 10.1016/j.clp.2018.05.011 30144846

[4] Ancel PY Livinec F Larroque B Marret S Arnaud C Pierrat V. Cerebral palsy among very preterm children in relation to gestational age and neonatal ultrasound abnormalities: the EPIPAGE cohort study. Pediatrics. (2006) 117:828–35. 10.1542/peds.2005-0091 16510664

[5] Himpens E Van den Broeck C Oostra A Calders P Vanhaesebrouck P. Prevalence, type, distribution, and severity of cerebral palsy in relation togestational age: a meta-analytic review. Dev Med Child Neurol. (2008) 50:334–40. 10.1111/j.1469-8749.2008.02047.x 18355333

[6] Linsell L Malouf R Morris J Kurinczuk JJ Marlow N. Prognostic factors for cerebral palsy and motor impairment in children born very preterm or very low birthweight: a systematic review. Dev Med Child Neurol. (2016) 58:554–69. 10.1111/dmcn.12972 26862030 PMC5321605

[7] Smith DD Sagaram D Miller R Gyamfi-Bannerman C. Risk of cerebral palsy by gestational age among pregnancies at-risk for preterm birth. J Matern Fetal Neonatal Med. (2020) 33:2059–63. 10.1080/14767058.2018.1536745 30318944

[8] Örtqvist M Einspieler C Ådén U. Early prediction of neurodevelopmental outcomes at 12 years in children born extremely preterm. Pediatr Res. (2021) 91:1522–9. 10.1038/s41390-021-01564-w 33972686

[9] Spittle AJ Orton J Anderson P Boyd R Doyle L. Early developmental intervention programs post hospital discharge to prevent motor and cognitive impairments in preterm infants. Cochrane Database Syst Rev. (2015) 12:CD005495. 10.1002/14651858.CD005495.pub4 17443595

[10] Bolk J Farooqi A Hafström M Ådén U Serenius F. Developmental coordination disorder and its association with developmental comorbidities at 6.5 years in apparently healthy children born extremely preterm. JAMA Pediatr. (2018) 172:765–74. 10.1001/jamapediatrics.2018.1394 29868837 PMC6142915

[11] Broström L Vollmer B Bolk J Eklöf E Ådén U. Minor neurological dysfunction and associations with motor function, general cognitive abilities, and behaviour in children born extremely preterm. Dev Med Child Neurol. (2018) 60:826–32. 10.1111/dmcn.13738 29573402

[12] Prechtl HFR Einspieler C Cioni G Bos AF Ferrari F. Spontaneous Motor Activity as a Diagnostic Tool. Demonstration Video. London; Graz: The GM Trust (1997).

[13] Prechtl HFR Einspieler C Cioni G Bos AF Ferrari F Sontheimer D. An early marker for neurological deficitis after perinatal brain lesioin. Lancet. (1997) 349:1361–3. 10.1016/S0140-6736(96)10182-3 9149699

[14] Einspieler C Prehtl HFR Ferrari F Cioni G Bos AF. The quantitive assessment of general movements in preterm, term and young infants – review of the methodology. Early Hum Dev. (1997) 50:47–60. 10.1016/S0378-3782(97)00092-3 9467693

[15] Bosanquet M Copeland L Ware R Boyd R. A systematic review of tests to predict cerebral palsy in young children. Dev Med Child Neurol. (2013) 55:418–26. 10.1111/dmcn.12140 23574478

[16] Novak I Morgan C Adde L Blackman J Boyd RN Brunstrom-Hernandez J. Early, accurate diagnosis and early intervention in cerebral palsy: advances in diagnosis and treatment. JAMA Pediatr. (2017) 171:897–907. 10.1001/jamapediatrics.2017.1689 28715518 PMC9641643

[17] Morgan C Crowle C Goyen TA Hardman C Jackman M Novak I. Sensitivity and specificity of general movements assessment for diagnostic accuracy of detecting cerebral palsy early in an Australian context. J Paediatr Child Health. (2016) 52:54–9. 10.1111/jpc.12995 26289780

[18] Einspieler C Marschik PB Milioti S Nakajima Y Bos AF Prechtl HFR. Are abnormal fidgety movements an early marker for complex minor neurological dysfunction at puberty? Early Hum Dev. (2007) 83:521–5. 10.1016/j.earlhumdev.2006.10.001 17129688

[19] De Bock F Will H Behrenbeck U Jarczok MN Hadders-Algra M Philippi H. Predictive value of general movement assessment for preterm infants' development at 2 years—implementation in clinical routine in a non-academic setting. Res Dev Disabil. (2017) 62:69–80. 10.1016/j.ridd.2017.01.012 28113095

[20] Spittle AJ Brown NC Doyle LW Boyd RN Hunt RW Bear M. Quality of general movements is related to white matter pathology in very preterm infants. Pediatrics. (2008) 121:e1184–9. 10.1542/peds.2007-1924 18390959

[21] Einspieler C Prechtl HFR Bos AF Ferrari F Cioni G. Prechtl's Method on the Qualitative Assessment of General Movements in Preterm, Term and Young Infants. London: Mac Keith Press (2008). 10.1016/s0378-3782(97)00092-3 9467693

[22] Hadders-Algra M. General movements: a window for early identification of children at high risk for developmental disorders. J Pediatr. (2004) 145 (2 Suppl):S12–8. 10.1016/j.jpeds.2004.05.017 15292882

[23] Zang FF Yang H Han Q Cao JY Tomantschger I Krieber M. Very low birth weight infants in China: the predictive value of the motor repertoire at 3 to 5months for the motor performance at 12months. Early Hum Dev. (2016) 100:27–32. 10.1016/j.earlhumdev.2016.03.010 27391870 PMC5010039

[24] Hitzert MM Roescher AM Bos AF. The quality of general movements after treatment with low-dose dexamethasone in preterm infants at risk of bronchopulmonary dysplasia. Neonatology. (2014) 106:222–8. 10.1159/000362919 25012880

[25] Soorani-Lunsing I Woltil HA Hadders-Algra M. Are moderate degrees of hyperbilirubinemia in healthy term neonates really safe for the brain? Pediatr Res. (2001) 50:701–5. 10.1203/00006450-200112000-00012 11726727

[26] Lunsing RJ Pardoen WF Hadders-Algra M. Neurodevelopment after moderate hyperbilirubinemia at term. Pediatr Res. (2013) 73:655–60. 10.1038/pr.2013.28 23407118

[27] Bhutta AT Cleves MA Casey PH Cradock MM Anand KJS. Cognitive and behavioral outcomes of school-aged children who were born preterm: a meta- analysis. JAMA. (2002) 288:728–37. 10.1001/jama.288.6.728 12169077

[28] van Iersel PA Bakker SC Jonker AJ Hadders-Algra M. Quality of general movements in term infants with asphyxia. Early Hum Dev. (2009) 85:7–12. 10.1016/j.earlhumdev.2008.05.006 18603385

[29] Wu YC Bouwstra H Heineman KR Hadders-Algra M. Atypical general movements in the general population: prevalence over the last 15 years and associated factors. Acta Paediatr. (2020) 109:2762–9. 10.1111/apa.15329 32335944 PMC7754433

[30] Bouwstra H Dijk-Stigter GR Grooten HMJ Janssen-Plas FEM Koopmans AJ Mulder CD. Prevalence of abnormal general movements in three-month-old infants. Early Hum Dev. (2009) 85:399–403. 10.1016/j.earlhumdev.2009.01.003 19211203

[31] Chang L Oishi K Skranes J Buchthal S Cunningham E Yamakawa R. Sex-Specific alterations of white matter developmental trajectories in infants with prenatal exposure to methamphetamine and tobacco. JAMA Psychiatry. (2016) 73:1217–27. 10.1001/jamapsychiatry.2016.2794 27829078 PMC6467201

[32] Wu YC Straathof E Heineman K Hadders-Algra M. Typical general movements at 2 to 4 months: movement complexity, fidgety movements, and their associations with risk factors and SINDA scores. Early Hum Dev. (2020) 149:105135. 10.1016/j.earlhumdev.2020.105135 32795785

[33] Örtqvist M Einspieler C Marschik P Ådén U. Movements and posture in infants born extremely preterm in comparison to term-born controls. Early Hum Dev. (2021) 154:105304. 10.1016/j.earlhumdev.2020.105304 33556691

[34] Kwong AKL Doyle LW Olsen JE Eeles AL Lee KJ Cheong JLY. Early motor repertoire and neurodevelopment at 2 years in infants born extremely preterm or extremely-low-birthweight. Dev Med Child Neurol. (2022) 64:855–62. 10.1111/dmcn.15167 35103304

[35] Thomas CW Meinzen-Derr J Hoath SB Narendran V. Neurodevelopmental outcomes of ELBW ventilated with continuous positive airway pressure vs mechanical ventilation. Indian J Pediatr. (2012) 79:218–23. 10.1007/s12098-011-0535-5 21853318 PMC3498084

[36] De Jesus LC Pappas A Shankaran S Li L Das A Bell EF. Outcomes of small for gestational age infants born at < 27 weeks' gestation. J Pediatr. (2013) 163:55–60.e1–3. 10.1016/j.jpeds.2012.12.097 23415614 PMC3947828

[37] Wilson-Costello D Friedman H Minich N Siner B Taylor G Schluchter M. Improved neurodevelopmental outcomes for extremely low birth weight infants in 2000-2002. Pediatrics. (2007) 119:37–45. 10.1542/peds.2006-1416 17200269

[38] Belfort MB Kuban KC O'Shea TM Allred EN Ehrenkranz RA Engelke SC. Weight status in the first 2 years of life and neurodevelopmental impairment in extremely low gestational age newborns. J Pediatr. (2016) 168:30–5.e2. 10.1016/j.jpeds.2015.09.036 26470687 PMC4698026

[39] Toome L Varendi H Männamaa M Vals MA Tänavsuu T Kolk A. Follow-up study of 2-year-olds born at very low gestational age in Estonia. Acta Pediatr. (2013) 102:300–7. 10.1111/apa.12091 23176138

[40] Constantinou JC Adamson-Macedo EN Mirmiran M Ariagno RL Fleisher BE. Neurobehavioral assessment predicts differential outcome between VLBW and ELBW preterm infants. J Perinatol. (2005) 25:788–93. 10.1038/sj.jp.7211403 16292337

[41] Vohr BR Stephens BE Higgins RD Bann CM Hintz SR Das A. Are outcome of extremely preterm infants improving? Impact of bayley assesment on outcomes. J Pediatr. (2012) 161:222–8.e3. 10.1016/j.jpeds.2012.01.057 22421261 PMC3796892

[42] Spittle AJ Spencer-Smith MM Cheong JL Eeles AL Lee KJ Anderson PJ. General movements in very preterm children and neurodevelopment at 2 and 4 years. Pediatrics. (2013) 132:e452–8. 10.1542/peds.2013-0177 23878041

[43] Nakajima Y Einspieler C Marschik PB Bos AF Prechtl HF. Does a detailed assessment of poor repertoire general movements help to identify those infants who will develop normally? Early Hum Dev. (2006) 82:53–9. 10.1016/j.earlhumdev.2005.07.010 16153788

[44] Bruggink JL Einspieler C Butcher PR Van Braeckel KN Prechtl HF Bos AF. The quality of the early motor repertoire in preterm infants predicts minor neurological dysfunction at school age. J Pediatr. (2008) 153:32–9. 10.1016/j.jpeds.2007.12.047 18571531

[45] Einspieler C Bos AF Libertus ME Marschik PB. The general movement assessment helps us to identify preterm infants at risk for cognitive dysfunction. Front Psychol. (2016) 22:406. 10.3389/fpsyg.2016.00406 27047429 PMC4801883

