# Supplementary material for: Identification of risk factors in pre-term infants with abnormal general movements
Source: Front Neurol. 2022 Nov 14;13:850877. doi: 10.3389/fneur.2022.850877 (PMC9701825; doi:10.3389/fneur.2022.850877)
Supplement: Supplementary file 1 [file Table_1.pdf]

## *Supplementary Material*

Table S1. The questionnaire of prenatal risk factors.

| PRENATAL RISK FACTORS | |
| --- | --- |
| **Maternal pregnancy risk factors** | – infertility treatments<br>– multiple pregnancies<br>– gynaecological operations<br>– untreated maternal hypothyroidism<br>– maternal acute viral/bacterial infections<br>– maternal other infections during pregnancy, such as toxoplasmosis<br>– rubella, cytomegalovirus, herpes |
| **Risk of a miscarriage** | – pregnancy poisoning (edema, EPH - gestosis, pregnancy throating, preeclampsia/eclampsia)<br>– bleeding during pregnancy<br>– short cervix<br>– premature labour contractions<br>– premature withdrawal of fetal water |
| **Predisposing intrauterine factors** | – fetal growth restriction,<br>– placental vascular disorders<br>– intrauterine infection/in ammation<br>– congenital anomalies of uterine, gynaecological and placental |

Table S2. The questionnaire of perinatal risk factors.

| PERINATAL RISK FACTOR | |
|---|---|
| **Kinds of childbirth and delivery methods** | – normal vaginal delivery<br>– fast delivery ("street")<br>– failure to progress (Prolonged Labor)<br>– cesarean delivery (C-section)<br>– management of complications during labour requires additional measures (eg, induction of labour, forceps or a vacuum-assisted delivery) |
| **Complications of labour and delivery** | – premature rupture of membranes<br>– abnormal levels of amniotic fluid: Oligohydramnios/ polyhydramnios<br>– intrauterine hypotrophy<br>– "nuchal cord" (umbilical cord becomes wrapped around the baby's neck, either once or multiple times)<br>– a true knot (umbilical cord becomes tied in a knot)<br>– meconium aspiration syndrome<br>– acute events at the peripartum period—placental abruption, chorioamnionitis, and birth asphyxia |
| **Childbirth complications** | – abnormal fetal presentation<br>– fetopelvic disproportion<br>– shoulder dystocia<br>– meconium aspiration syndrome<br>– fever, untreated jaundice, hyperbilirubinemia (above 15 mg %)<br>– prolonged artificial respiratory ventilation, neonatal resuscitation |

Table S3. The questionnaire of postnatal risk factors.

| POSTNATAL FACTOR RISK | |
|---|---|
| **Neonatal complication** | – birth asphyxia (BA)<br>– disturbances of muscle tone (i.e. hypothermia, muscle tremor, lethargy, weak sucking reflex)<br>– repeated neonatal convulsions (Seizures)<br>– acute organ infections (eg. pneumonia, diarrhoea, anaemia, and urinary infections)<br>– high bilirubin levels<br>– respiratory distress syndrome (RDS)<br>– periventricular leukomalacia (PVL)<br>– intraventricular haemorrhage IVH grades 3-4 (confirmed by USG)<br>– bronchopulmonary dysplasia (BPD)<br>– sepsis/meningitis |
